# Recent Advances in Phenylboronic Acid-Based Gels with Potential for Self-Regulated Drug Delivery

**DOI:** 10.3390/molecules24061089

**Published:** 2019-03-19

**Authors:** Chenyu Wang, Bozhong Lin, Haopeng Zhu, Fei Bi, Shanshan Xiao, Liyan Wang, Guangqing Gai, Li Zhao

**Affiliations:** 1 Department of Orthopedics, Hallym University, 1 Hallymdaehak-gil, Chuncheon, Gangwon-do 200-702, Korea; cathywang0111@hotmail.com; 2 Laboratory of Building Energy-Saving Technology Engineering, College of Material Science and Engineering, Jilin Jianzhu University, Changchun 130118, China; bzlin2018@163.com (B.L.); hpzhu2018@163.com (H.Z.); bifei1224@163.com (F.B.); xiaoshanshan@jlju.edu.cn (S.X.); gaigq@163.com (G.G.)

**Keywords:** phenylboronic acid, gel, glucose sensitivity, drug delivery, diabetes therapy

## Abstract

Glucose-sensitive drug platforms are highly attractive in the field of self-regulated drug delivery. Drug carriers based on boronic acid (BA), especially phenylboronic acid (PBA), have been designed for glucose-sensitive self-regulated insulin delivery. The PBA-functionalized gels have attracted more interest in recent years. The cross-linked three-dimensional (3D) structure endows the glucose-sensitive gels with great physicochemical properties. The PBA-based platforms with cross-linked structures have found promising applications in self-regulated drug delivery systems. This article summarizes some recent attempts at the developments of PBA-mediated glucose-sensitive gels for self-regulated drug delivery. The PBA-based glucose-sensitive gels, including hydrogels, microgels, and nanogels, are expected to significantly promote the development of smart self-regulated drug delivery systems for diabetes therapy.

## 1. Introduction

Diabetes mellitus threatens human health seriously with a series of complications, such as cardiovascular disease [1]. The worldwide prevalence of diabetics is predicted to be about 366 million in 2030 with increasing attention for the treatment of diabetes [2]. The frequent injection of exogenous insulin is the major treatment of diabetes. In spite of quickly decreasing the blood glucose level, frequent insulin injection causes inevitable injection suffering and decline of the quality of life of patients. Even though implantable insulin pumps are the current optimal therapy for type 1 diabetic patients, their high cost limits their wide clinical application [3]. Smart insulin modified with an aliphatic domain and a phenylboronic acid (PBA) has been developed to tune the pharmacokinetics of insulin activity for personalized therapy, however, it must interface with insulin pumps, infusion devices, or controlled release materials to further improve performance [4]. An alternative treatment of diabetes with continuous and automatic regulation of drug release triggered by glucose directly is required. Therefore, glucose-sensitive materials, a kind of “intelligent” polymer, have greatly been used in self-regulated systems. Self-regulated systems, which are called an artificial pancreas, control insulin release triggered by elevated level of blood glucose continuously and automatically. By integrating glucose-triggered drug delivery with minimal patient intervention and improved diabetic life quality, glucose-sensitive drug delivery systems may prove valuable in diabetes therapy and replace frequent insulin injection [5].

Three kinds of glucose-sensitive materials have attracted growing scientific attention and are considered novel candidates to serve as self-regulated drug delivery systems. One strategy is based on glucose oxidase (GOD), which converts glucose to gluconic acid upon a pH change of the microenvironment, resulting in physicochemical changes of GOD-incorporated carriers [6]. The physicochemical changes of GOD-incorporated platforms induce glucose-triggered payload release [7,8,9,10]. Concanavalin A (Con A) is also be used to design glucose-sensitive drug delivery systems due to the specific binding capacity for glucose, glycopolymers, and polysaccharides [11,12,13,14]. However, GOD and Con A are protein-based components, and the instability and biotoxicity of GOD and Con A during fabrication and storage restrict their application in self-regulated drug delivery [15,16].

**Scheme 1:**
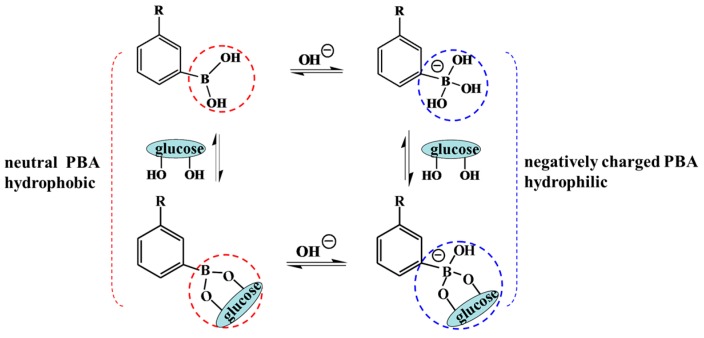
Complexation equilibrium between phenylboronic acid (PBA) and glucose.

An alternative mechanism based on totally synthetic components, boronic acid (BA) and especially PBA, and their derivatives, have been largely investigated for self-regulated drug delivery systems [17,18,19,20]. Since Okano and coworkers firstly reported PBA-based glucose-triggered drug release, PBA and its derivatives have been greatly employed to design glucose-triggered drug delivery systems [21,22]. PBA-containing platforms are much more stable and suitable for long-term storage than GOD- and Con A-based drug carriers [23]. The principle of the PBA-mediated glucose-sensitive drug delivery system is based on the reversible reaction between PBA and *cis*-diol compounds [24]. There are two states of PBA in aqueous solution. One is the neutral trigonal-planar form and the other is the negatively charged tetrahedral boronate form. Between the two states of PBA moieties there is an equilibrium (Scheme 1). When in aqueous solution with pH above the p*K*_a_ of PBA (i.e., 8.2−8.6), most PBA moieties are negative and relatively hydrophilic. However, the PBA moieties are neutral and hydrophobic when the pH of the solution is below the p*K*_a_ of PBA [25,26]. In the presence of glucose or other 1,2- or 1,3-diols, both kinds of PBA moieties form a 5- or 6-cyclic boronic ester. However, the cyclic boronic ester between neutral trigonal-planar PBA and a diol cannot be formed with easy hydrolysis. The negatively charged PBA state forms a more stable cyclic boronic ester with *cis*-diol compounds, resulting in the increase of the negatively charged PBA content and improved hydrophilicity of PBA-containing materials [27,28]. The increased hydrophilicity of PBA-modified materials induces the swelling, disassembly, and/or destruction of PBA-mediated platforms with subsequent release of the payload. Glucose can also form bis-bidendate complexes with PBA resulting from the reaction between one glucose molecule and two cis-diols. Therefore, the bis-bidendate complexes induce the shrinking of PBA-functionalized platforms [29]. All the swelling and shrinking of platforms alter the diffusion behavior of the payload, triggered by glucose-sensitive volume phase transition. PBA-functionalized materials present great potential applications in self-regulated drug delivery [29,30].

There are many forms of PBA-functionalized platforms which have been exploited to investigate glucose sensitivity. Polymer micelles, vesicles, and capsules are obtained by the self-assembly of PBA-modified amphiphilic polymers, while layer-by-layer (LbL) films are obtained by the alternating adsorption of polymers with opposite charges. Even though these devices have good glucose sensitivity, the concentration-dependent disintegration, unstably long-term storability, and weakened mechanical strength restrict their applications in glucose-sensitive drug delivery. PBA-functionalized mesoporous silica nanoparticles (MSN) have attracted improving interests due to their characteristics of high biocompatibility, improved dug loading, and functionalized surface. The glucose-sensitive nanocarriers and LbL films and microcapsules for self-regulated drug delivery have been reviewed [26,31]. Besides, PBA-based hydrogels, microgels, and nanogels with chemically or physically cross-linked structures have great potential applications in self-regulated drug delivery systems. The 3-D structures endow the hydrogels, microgels, and nanogels with reversible physicochemical changes that result in payload release. Usually, hydrogels are macroscopic hydrogels. Microgels are microsized hydrogel particles, while nanogels are hydrogel nanoparticles or nanohydrogels with nano-scale dimensions (typically 20~250 nm) [32,33]. The Zhang group and Catargi group have discussed the fabrication and application of PBA-containing macroscopic hydrogels and microgels with discussion of their further development [29,34]. However, studies of PBA-based hydrogels, microgels, and nanogels for self-regulated drug delivery are rare. In summary, this article reviews the recent development of self-regulated drug delivery based on PBA-mediated glucose-sensitive gels.

## 2. PBA-Based Hydrogels

Hydrogels have received considerable attention for drug delivery applications. There have been extensive studies on PBA-functionalized hydrogels for self-regulated drug delivery systems.

Low-molecular-weight hydrogels (LMWG) have been largely investigated for controlled drug delivery due to their super-sensitivity to external stimuli. LMWG were formed by the self-assembly of gelators with low-molecular-weight, where the gel-sol transition was easily achieved by changing stimuli. An injectable glucose-sensitive LMWG was prepared by the self-aggregation of the gelator which was synthesized with a pyrene moiety coupled with PBA by L-phenylalanine and a 2,2′-(ethylenedioxy) bis(ethylamine) linker [35]. The pyrene unit and PBA moiety endowed the physical cross-linked LMWG with glucose sensitivity, and the resulting compound was utilized to detect glucose and control insulin release at physiological pH. Differently from chemically cross-linked hydrogels, physically cross-linked networks have transient junctions arising from hydrogen bonding, π–π stacking, and van der Waals interactions. The self-aggregation of the gelator provides a new strategy for the design of PBA-based glucose-sensitive hydrogels.

**Figure 1:**
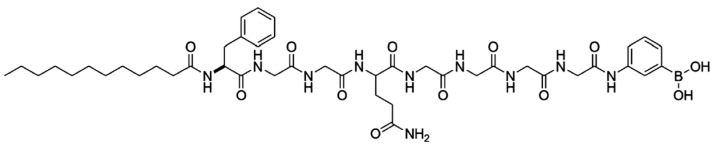
The structure of the gelator used for self-regulated drug delivery. (Reprinted from ref. [38]).

Gao and coworkers investigated the dual-responsiveness of a PBA-based low-molecular-weight organogel to both glucose and pH [36]. In addition, the group studied the gelation properties and glucose sensitivity of a PBA-containing low-molecular-weight organogel based on an alkyl chain (C2–C11), where the glucose sensitivity of the organogel was associated with the molecular structure of the gelator [37]. Integrating an oligopeptide with PBA, the same group demonstrated that the LMWG self-assembled from a gelator exhibited dual-responsive and long-lasting drug delivery [38]. The structure of the gelator is shown in Figure 1. The hydrophilicity of gelator was regulated by the oligopeptide which was made of the natural amino acids L-phenylalanine, glycylglycine, and L-glutamine. The alkyl chain of lauroyl chloride was introduced to adjust the hydrophobicity of the gelator in favor of the sol-gel translation. PBA unit endowed the gelator with glucose sensitivity. When the sol was cooled to body temperature, LMWG was obtained very quickly within two minutes. The sol-gel translation resulted from the enhanced π–π stacking owing to the benzene units as well as hydrogen bonding and van der Waals force within the gelators. The LMWG was very stable during the drug release even for three months or with sever shaking, indicating its long-lasting drug delivery. The pH-sensitivity of the hydrogels, a result of the ionization of the PBA and amide moieties in acid or alkali solutions, was used for doxorubicin delivery for cancer therapy. Importantly, PBA endowed the hydrogels with glucose sensitivity. The release of phenformin, an antidiabetic drug, was regulated in response to changes in glucose concentration. The fact that increasing glucose concentration induced much more drug release indicated its promising application in a self-regulated drug delivery system. Integrating the pH-sensitivity with glucose-triggered drug release, the long-lasting glucose/pH-responsive hydrogels will have notable importance in the development of self-tuning controlled-release systems for diabetes and cancer therapy.

Even though LMWG used as a drug carrier possesses good glucose-sensitive drug delivery, the stability of LMWG is not sustainable enough for long-term drug delivery compared to the supramolecular hydrogels with chemical cross-linked structure. A multiresponsive hydrogel was synthesized by copolymerization of (2-dimethylamino) ethyl methacrylate (DMAEMA) and 3-acrylamidephenylboronic acid (AAPBA) using *N*,*N*′-methylenebisacrylamide (NNMBA) as a cross-linker [39]. The P(DMAEMA-*co*-AAPBA) interpenetrating (IPN) hydrogels possessed glucose-triggered drug release under physiological pH and temperature. The increased Lewis acidity of the boron center lowered the p*K*_a_ of the PBA residues. The Lewis acid–base interactions between the electron-poor boron atom and the electron-rich nitrogen atom on poly(DMAEMA) endowed the hydrogel with glucose sensitivity under physiological pH. These multiresponsive hydrogels have important application in self-regulated drug delivery.

Also based on poly(DMAEMA), a novel triple-responsive semi-interpenetrating (semi-IPN) hydrogel was exploited [40]. Poly(3-acrylamidephenylboronic acid-*co*-(2-dimethylamino) ethyl methacrylate) (P(AAPBA-*co*-DMAEMA)) has a cross-linked structure incorporated with interpenetrating β-cyclodextrin-epichlorohydrin (β-CD-EPI)—P(AAPBA-*co*-DMAEMA)/(β-CD-EPI) semi-IPN hydrogel. The higher content of β-CD decreased the equilibrium swelling ratios (ESRs) of semi-IPN hydrogels due to the complexation between PBA groups and the dihydroxyl of β-CD. Besides, pH, temperature, ionic strength, and glucose concentration of the media significantly affected the ESRs of the hydrogels. Using ibuprofen and aminophylline as hydrophobic and hydrophilic model drugs, the drug loading and release profiles were investigated. The structure of the hydrogels affected the drug loading ratio. Increased β-CD content led to of ibuprofen and a lower drug loading ratio of aminophylline. The lower drug loading ratio of hydrophilic aminophylline in hydrogels with high β-CD content resulted from the lower ESRs, which was induced by the increased complexation between PBA and β-CD. However, the loading ratio of hydrophobic ibuprofen increased with the increase of β-CD content in hydrogels. The reason was that hydrophobic ibuprofen formed inclusion complexes with β-CD via host–guest interactions. The release profiles of drug from the hydrogels could be adjusted by pH, temperature, glucose concentration, and type release medium. The semi IPN hydrogels may be useful as a guideline for the optimal design of glucose-sensitive drug delivery systems.

To enhance the response of PBA-based hydrogel to blood glucose concentration, a comb-type grafted poly(*N*-isopropylacrylamide-*co*-3-acrylamidophenylboronic acid) (poly(NIPAM-*co*-AAPBA)) hydrogel was exploited [41]. The hydrogel was introduced by grafting poly(NIPAM-*co*-AAPBA) side chains onto cross-linked poly(NIPAM-*co*-AAPBA) networks. The comb-type hydrogel presented a rapid response to the blood glucose concentration owing to the effects of the freely mobile ends of the grafted poly(NIPAM-*co*-AAPBA). Grafted poly(NIPAM-*co*-AAPBA) side chains formed complexes with glucose quickly without any restrictions than that between cross-linked poly(NIPAM-*co*-AAPBA) hydrogel and glucose. The rapid response rate endowed the grafted hydrogel with attractive applications in self-regulated drug delivery systems.

Besides the structure of hydrogels, the composition of hydrogels also affects the glucose sensitivity. Magda and coworkers studied the effect of chemical composition on the response of zwitterionic glucose-sensitive hydrogels using DOE (design of experiments) methods [42]. Zwitterionic glucose-sensitive hydrogels were prepared by the copolymeration of *N*-(3-(dimethylamino)propyl) acrylamide (DMAPAA) and AAPBA. The molar ratio of AAPBA/DMAPAA and the wt % of the monomer in the pregel solution were the primary factor for determining the value of the inverse of the 1st order rate constant, where a decreasing amount of cross-linker obtained faster glucose responses.

**Figure 2:**
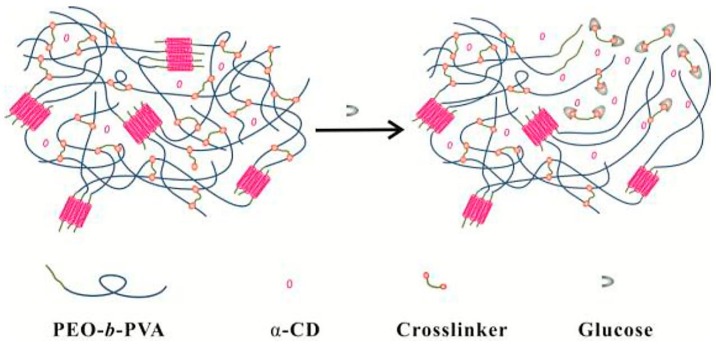
Schematic and the glucose-sensitive mechanism of the hydrogel composed of PEO-*b*-PVA, *α*-CD, and double PBA-terminated poly (ethylene oxide) (PEO) cross-linker. (Reprinted from ref. [45].)

In addition to stable chemical cross-linked hydrogels using a small molecular as the cross-linker, reversible dynamic cross-linked hydrogels have also been designed as glucose-sensitive drug carriers. Hydrogels were obtained by the reversible covalent complexation of PBA with *cis*-1,2 or *cis*-1,3-diol compounds as a dynamic link [43,44]. Glucose-sensitive solid-like hydrogels based on dynamic covalent chemistry and inclusion complexation were also designed [45]. By simply mixing the solutions of poly (ethylene oxide)-*b*-poly vinyl alcohol diblock polymer (PEO-*b*-PVA), α-cyclodextrin (*α*-CD), and a double PBA-terminated PEO cross-linker, hydrogels were easily obtained. As shown in Figure 2, the inclusion complexation between PEO and *α*-CD, and the dynamic covalent bonds between PBA and PVA, strengthened the hydrogel network. In addition, the formation of hydrogels was the cooperative interaction of the inclusion complexation and dynamic covalent chemistry. The increase of *α*-CD content shortened the gelation time and enhanced the structural recovery ability with the significant contribution of increased dynamic covalent chemistry to the cross-linking density of hydrogel network. The release of FITC-labeled BSA from the hydrogels had no burst release property at physiological pH, indicating that the FITC-labeled BSA was loaded inside the hydrogels instead of being adsorbed on the surfaces of the hydrogels. These hydrogels have great potential as self-regulated drug delivery vehicles with tunable glucose sensitivity.

**Figure 3:**
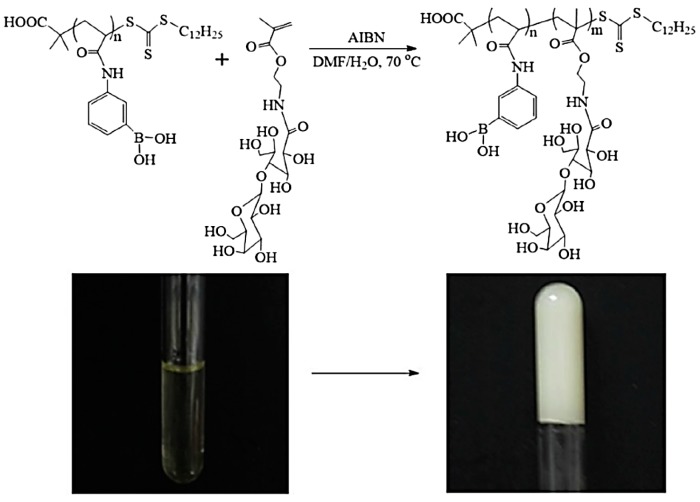
The formation of PBA-containing glycopolymer hydrogel before and after. (Reprinted from ref. [46]).

Via dynamic boronic ester bonds, glycopolymer hydrogels were exploited to control insulin release triggered by glucose [46]. The copolymerization of AAPBA and 2-lactobionamidoethyl methacrylate (LAMA) was conducted by the reversible addition–fragmentation chain transfer (RAFT) method and the obtained block glycopolymer formed hydrogels by phenylboronate-diol crosslinked binding (Figure 3). The LAMA content played an important role in the swelling of the hydrogels. For the hydrogels with the highest LAMA content, the equilibrium swelling ratio was up to 1856%. In addition, with high LAMA content, the insulin loading capacity of the hydrogels increased. Higher content of LAMA enhanced the charged phenylborates, which resulted in enhanced hydrophilicity of the hydrogels and subsequently promoted drug permeation and adsorption. The introduction of carbohydrate moieties improved the cytocompatibility of the glycopolymer hydrogels. The insulin release from the glycopolymer hydrogels exhibited glucose sensitivity due to glucose-induced dissociation of boronic ester linkages of hydrogels. Importantly, the released insulin possessed an original conformation compared with standard insulin. PBA-based glycopolymer hydrogels are an alternative design of glucose-sensitive drug delivery systems.

**Figure 4:**
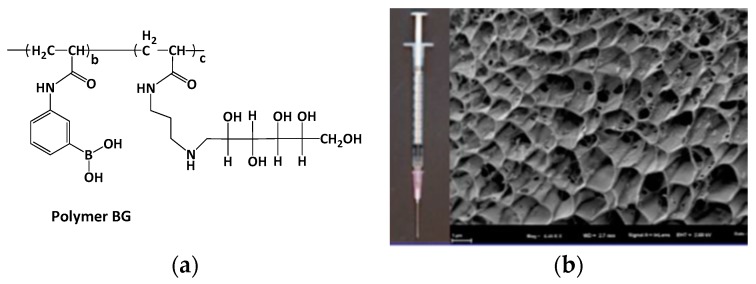
(**a**) Structure of PBA-containing glycopolymer BG; (**b**) the soft and injectable hydrogel formed by polymer BG with 50% PBA, and the SEM image of the obtained hydrogel. Scale bar: 1 μm. (Reprinted from ref. [47]).

Another series of glucose-sensitive block glycopolymer hydrogels based on dynamic boronic ester bonds were exploited for glucose-sensitive drug delivery [47]. As shown in Figure 4a, block glycopolymer, (3-propionamidophenyl)boronic acid (*N*-(3-((2,3,4,5,6-pentahydroxyhexyl)amino)propyl)propionamide) (noted polymer BG), was cross-linked through PBA–glucose complexation within the glycopolymer and injectable self-healing hydrogels were obtained. The glycopolymer with 10–60% content of PBA formed self-supporting hydrogels at 5% weight in water, which could be easily loaded and extruded through a needle with the reformation of hydrogels (Figure 4b). The hydrogels possessed rapid recovery with shear-thinning and self-healing behaviors which were consistent with the injectable hydrogel. In addition, the glycopolymer hydrogels exhibited glucose-triggered rhodamine B release. Integrating injectable and self-healing properties with glucose-induced drug release, glycopolymer hydrogels have potential application in the treatment of diabetes.

**Figure 5:**
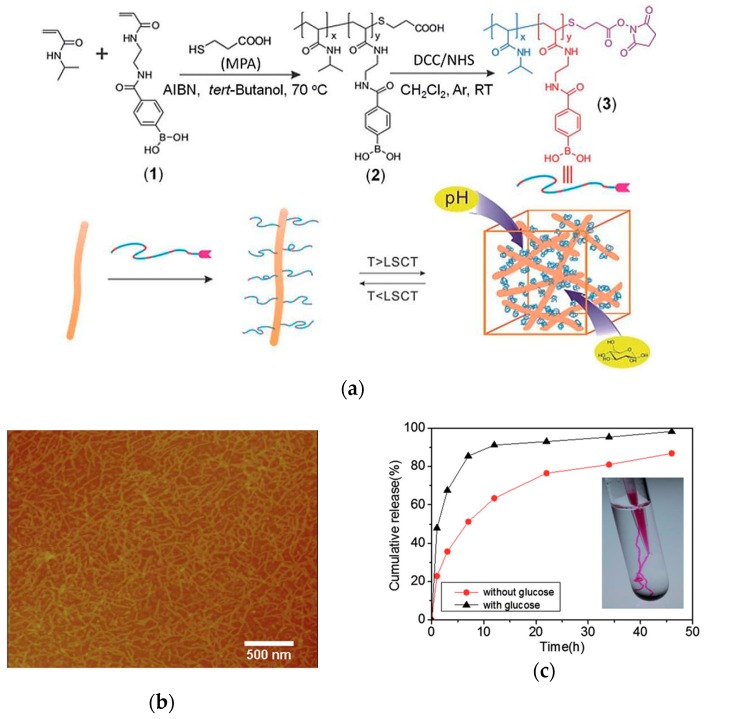
(**a**) Schematic representation of the preparation of the hybrid virus–polymer bioconjugate of end-functionalized PBA- and *N*-isopropylacrylamide (NIPAM)-containing copolymer to the rod-like M13 virus, and the multiresponsiveness of the hybrid virus–polymer bioconjugate; (**b**) Internal structure of the gel as revealed by AFM; (**c**) insulin release behavior of the virus based hydrogel in the presence or absence of glucose. Inset in (**c**) hydrogel forms instantly when the polymer grafted virus in the sol state was injected into the aqueous solution at 37 °C. (Reprinted from ref. [48]).

Hydrogels based on PBA-containing polymers for self-regulated drug delivery system have been studied widely, while the bioconjugates of PBA-functionalized polymer and protein or peptide have been rarely used for glucose-sensitive drug delivery. Multiresponsive hydrogels were prepared from the bioconjugates of end-functionalized PBA containing copolymers and rod-like M13 viruses [48]. The end-functionalized PBA containing copolymer poly(NIPAM-*co*-PBA)-NHS was synthesized by two steps (Figure 5a). Firstly, poly(NIPAM-*co*-PBA)-COOH was synthesized by chain transfer free radical copolymerization of NIPAM and 4-(1,6-dioxo-2,5-diaza-7-oxamyl) phenylboronic acid (DDOPBA) using 3-mercaptopropionic acid (MPA) as the chain transfer agent. Then, the −COOH of the obtained poly(NIPAM-*co*-PBA)-COOH was transferred into *N*-hydroxysuccinidic ester. The hybrid virus–polymer bioconjugate was prepared by the conjugation of poly(NIPAM-*co*-PBA)-NHS to rod-like M13 virus, which was a natural protein assembly with a large amount of functional groups. The gelation behavior of virus–polymer bioconjugates was multiresponsive and reversible. The gelation of virus–polymer bioconjugates was owing to the collapsed hydrophobic state of poly(NIPAM-*co*-PBA), which conferred the attractive interactions between the viruses and drove the viruses into the hydrogels. AFM revealed that the rod-like virus was interconnected with itself inside the hydrogel with large pores (Figure 5b). The virus–polymer bioconjugates were in sol state when the temperature was below the critical gelation temperate (*T*_g_), and in the gel state when the temperature was above *T*_g_. The *T*_g_ of virus–polymer bioconjugates was sensitive to pH. At pH 7.10, the *T*_g_ was 15 °C, and increased when the pH increased. Furthermore, the *T*_g_ of the hybrid virus–polymer bioconjugates was 18 °C in the absence of glucose, while it was 23 °C in the presence of glucose at pH 7.65. In addition, the negligible hysteresis for the sol-to-gel and opposite gel-to-sol transition cycles indicated the reversible gelation behavior of the virus–polymer bioconjugates. Besides, the temperature-sensitive gelation behavior was in favor of the encapsulation of bioactive species inside the hydrogels with high loading capacity. Insulin-loaded hydrogels were obtained by the injection of a mixture of insulin and polymer-grafted virus at 4 °C into PBS buffer at 37 °C (inset of Figure 5c). Glucose-sensitive insulin release was also observed (Figure 5c). Glucose-triggered insulin release, combined with reversible temperature-, pH-, and glucose-regulated gelation behavior, endowed the PBA-containing polymer-virus bioconjugates with great potential in self-regulated drug delivery.

Cross-linking glucose-sensitive hydrogels based on PBA provides suitable semiwetting with reversible swelling/shrinking changes which are suitable for self-regulated drug delivery. However, the glucose sensitivity of bulk hydrogels hysteretically results from the slow permeation of glucose into the hydrogel network. In addition, the practical application of bulk hydrogels for the regulation of blood glucose levels is restricted by the implantation or other surgical approach. Even though injectable hydrogels are convenient to administrate, a lot of effort is needed to promote the practical application of glucose-sensitive hydrogels for diabetes therapy.

## 3. PBA-Functionalized Microgels

Even though PBA-based hydrogels are glucose-sensitive, the implantation administration of bulk hydrogels and the inconvenient injection of injectable hydrogels restrict the practical application of glucose-sensitive hydrogels in self-regulated drug delivery. Compared with hydrogels, PBA-based microgels have promising application in glucose-sensitive drug delivery [49]. Microgels are gel particle dispersions with average diameters ranging between 50 nm and 5 mm [50]. The small size endows the microgels with rapid swelling or shrinking in response to environmental changes. The glucose-response time of PBA-containing microgels are much faster than that of hydrogels. Kataoka and coworkers reported that gel beads containing AAPBA and NIPAM need a very long time (as long as 400 min) for glucose sensitivity [51]. However, microgels with the same components need only 10^2^ s for glucose-induced swelling [52]. Besides faster glucose-response time, the administration of microgels using a minimally invasive method is another advantage of microgels for self-regulated drug delivery. In addition, microgels display high stability, which is promising for glucose-sensitive drug delivery vehicles.

A range of PNIPAM-based and PBA-functionalized microgels have been synthesized via the copolymerization of NIPAM and PBA-containing comonomers or the postpolymerization of functional PBA derivatives to PNIPAM microgels [53,54,55,56,57]. Poly(*N*-isopropylacrylamide-*co*-acrylic acid) P(NIPAM-*co*-AA) was modified by 3-aminophenylboronic acid (3-APBA), resulting in PBA-mediated glucose-sensitive microgels [58,59].

Glucose-induced swelling of the poly(*N*-isopropylacrylamide-*co*-3-acrylamidophenylboronic acid) (P(NIPAM-3-AAPBA)) microgel was explained by the formation of a 1:1 glucose–phenylboronate complex, which increased the degree of ionization and created a Donnan potential between the gel phase and the liquid phase [52,60].

**Figure 6:**
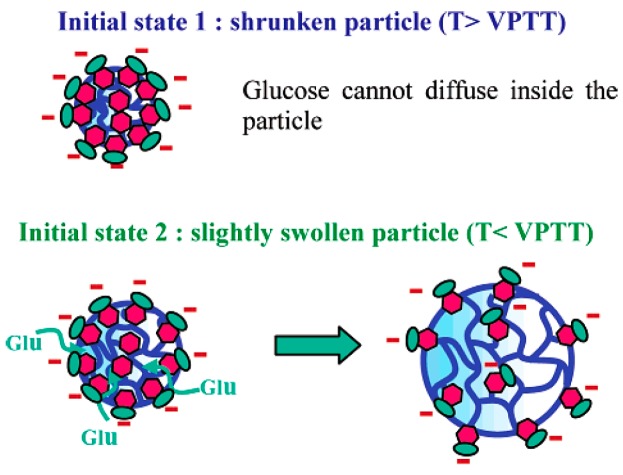
Schematic representation of microgel swelling upon glucose addition, showing the importance of the initial state. (Reprinted from ref. [61]).

For P(NIPAM-PBA) microgels, the swelling response of microgels to glucose relates to the microgel composition and glucose concentration. Ravaine studied the effects of PBA content and glucose concentration on the swelling of monodispersed PNIPAM submicrometric microgels modified with AAPBA at different temperatures [61]. Compared to native PNIPAM particles, the incorporation of AAPBA decreased the volume phase transition temperature (VPTT) of the P(NIPAM-PBA) microgels. With the increase of PBA content, the swelling response of the microgels increased. However, the swollen state of the microgels that was achieved with same PBA content was strongly dependent on the initial temperature of the suspension with a constant glucose concentration. As shown in Figure 6, when the initial temperature was above VPTT, the microgels were smaller and the swelling was slight. In this case, glucose was unable to diffuse inside the microgel particle when it was initially collapsed. Only a small part of glucose linked to the PBA which was incorporated on the surface of microgels. However, when the initial temperature was below VPTT, a slight swelling of the microgels was aided the permeation and diffusion of glucose to the inside of the microgel particle. As a result, all the PBA was linked by glucose to form more hydrophilic boronic ester, inducing a much higher swelling degree of the microgels.

Zhang and coworkers also studied the volume phase transitions of glucose-sensitive PBA-functionalized P(NIPAM-3-AAPBA) microgels which were synthesized by the coupling of 3-APBA to P(NIPAM-*co*-AA) microgels [62]. The microgels presented a two-stage thermosensitive volume phase transition in the presence of glucose. When temperature increased, the microgels underwent a small degree of collapse and a large volume change followed. In addition, the glucose sensitivity of the microgels was affected by pH. At pH = 7.5, which is below the *p*K_a_ of PBA, the glucose-induced size changes of the microgels were negligible. In this case, most PBA groups were hydrophobic with few boronate esters produced. However, when the pH increased, the glucose sensitivity of the microgels was enhanced as a result of the improved hydrophilicity of the microgels with resultant boronate ester. The glucose-induced size expansion of the microgels was depressed by high ionic strength due to the weakening of Donnan potential. At room temperature, remarkable glucose-induced swelling was observed in the microgels with high content of PBA.

**Figure 7:**
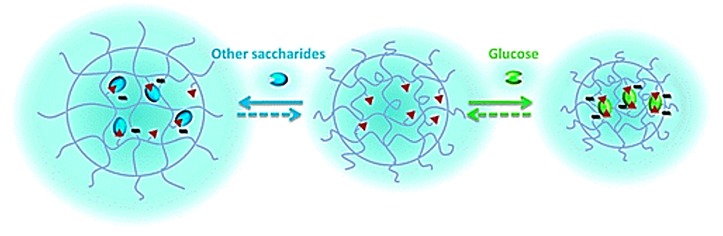
Schematic representation of glucose-induced microgels shrinking with glucose–bis(boronate) complexation (Reprinted from ref. [66]).

Most PBA-based microgels exhibit glucose-induced expansion due to the production of glucose–mono(boronate) complexes [57,63]. The glucose–mono(boronate) complexes make the ionization equilibrium of PBA shift from uncharged and hydrophobic state to the charged and hydrophilic state with the formation of tetrahedral boronate esters. The tetrahedral boronate esters further increase the hydrophilicity of the microgels and induce the swelling of microgels due to the glucose-induced increase in the Donnan potential. Besides glucose-induced swelling of microgels, glucose-induced shrinking of microgels also has been reported. As shown in Figure 7, glucose exhibits the unique property to form glucose–bis(boronate) complexes through its furanose form [64,65,66]. However, most physiological relevant saccharides, such as galactose, mannose, and fructose, can only bind to boronic acid as monodentate complexes [67]. Zhang designed a poly(N-isopropylacrylamide-*co*-2-acrylamidophenylboronic acid) (P(NIPAM-2-AAPBA)) microgel, which was synthesized by modification of the poly(*N*-isopropylacrylamide-*co*-acrylic acid) (P(NIPAM-AAc)) microgel with 2-aminophenylboronic acid (2-AAPBA) [17]. In the presence of glucose, the size of the P(NIPAM-2-AAPBA) microgels decreased, showing contraction-type glucose sensitivity. The glucose-induced shrinking of the microgels was related with the glucose–bis(boronate) complexes in which one glucose molecule complexed with two PBA groups in a 1:2 binding model. The resultant glucose–bis(boronate) complexes increased the cross-linking density and reduced the degree of swelling of the microgels. The contraction-type glucose-sensitive microgels offer a new strategy for the design of glucose-sensitive microgels.

Except for contraction-type glucose-sensitive microgels, glucose-induced shrinking/swelling microgels were also designed. Ravaine and coworkers firstly designed the PBA-containing microgels cross-linked with bis-boronate complexes which either swelled or shrank selectively depending on glucose concentration under physiological conditions [66]. The microgels were obtained by copolymerization of DDOPBA with an alkylacrylamide (NIPAM, *N*-isopropylmethacrylamide (NIPMAM), or *N*-ethylmethacrylamide (NEMAM)) and a cross-linking agent (*N*,*N′*-methylenebis(acrylamide) (BIS), or ethylene glycol dimethacrylate (EGDMA)). The effect of monomer composition on the swelling/shrinking behavior of the microgels was studied. For microgels based on NIPAM and PBA (P(NIPAM-PBA) microgels), the VPTT was associated with the PBA content. More PBA content lowed the VPTT of P(NIPAM-PBA) microgels, which was conformed in the author’s earlier work [61]. However, high glucose concentration increased the VPTT of P(NIPAM-PBA) microgels. In addition, at constant PBA content, the VPTT of NIPAM microgels was lower than that of NIPMAM microgels, which was lower than that of NEMAM microgels. All kinds of microgels displayed glucose-sensitive behaviors. When at a pH above the p*K*_a_ of PBA, EGDMA cross-linked NEMAM and NIPMAM microgels exhibited a two-step behavior wherein low glucose concentration induced microgel shrinkage and high glucose concentration induced microgel swelling. The shrinking behavior was associated with the additional cross-linking junctions due to glucose-bis(boronate) complexes at low glucose concentration. When the glucose concentration increased, the glucose–bis(boronate) complexes were converted to glucose–mono(boronate) complexes owing to the glucose-induced cross-link disruption. As a result, the microgels swelled under high glucose concentration due to the increased polymer charges and hydrophilicity. The glucose-response behavior of the microgels was related to the nature of the cross-linker and the modification of the PBA. The shrinking/swelling behavior was selective for glucose because the saccharide–bis(boronate) complexes were highly selective for glucose and could not occur with other sugars, such as fructose, which was also demonstrated by Asher and coworkers [67].

**Figure 8:**
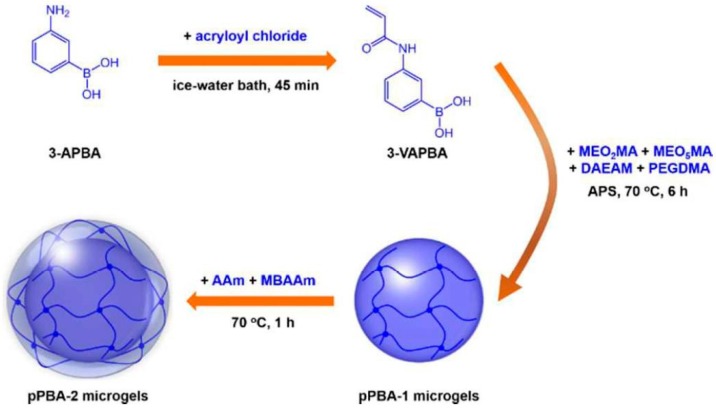
Illustration of the synthesis of the proposed pPBA microgels. (Reprinted from ref. [68]).

As mentioned above, polymers based on NIPAM, NEMAM, and EGDMA are thermosensitive and the VPTT of P(NIPAM-PBA) microgels are adjusted by glucose concentration and PBA content. Some other kinds of PBA-based microgels also possess volume phase transition behavior. The glucose-responsive volume phase transition behavior of poly(phenylboronic acid) (pPBA) microgels was switchable [68]. The preparation of the pPBA microgels was comprised 3-VAPBA covalently bonded onto the microgels of oligo(ethylene glycol)-based polymers (Figure 8). The stability of the pPBA-2 microgels was improved by further addition of a poly(acrylamide) (poly(AAm)) gel layer onto pPBA-1 microgels. In the presence of glucose, the glucose-response behaviors of pPBA microgels were different under different temperatures. At lower temperatures below 29.0 °C, the microgels shrunk when glucose was added, while at higher temperatures above 33.0 °C, the microgels swelled when glucose was added. Around 31.0 °C, negligible volume change of the microgels was recorded upon adding glucose. The pPBA microgels with switchable glucose-responsive volume phase transition behavior provide guidelines for the design of glucose-sensitive microgels.

**Figure 9:**
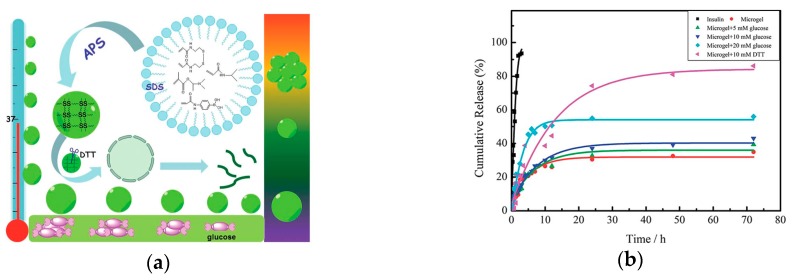
(**a**) Schematic illustration of smart multifunctional microgels with pH-, temperature-, and glucose sensitivity at physiological conditions and gradual degradation; (**b**) Release profiles of microgels in the presence of glucose in PBS of 7.4 at 37 °C. (Reprinted from ref. [69]).

Another multifunctional microgel with high stability and degradability was designed [69]. As shown in Figure 9a, the microgels were prepared by free radical polymerization of NIPAM, DMAEMA, and AAPBA through a precipitation emulsion method using reductive degradable *N,N′*-bis(arcyloyl)cystamine (BAC) as the cross-linker. The as-synthesized microgels exhibited pH-, temperature-, and glucose sensitivity at physiological conditions and gradual degradation. The porous network structure of the microgels was in favor of the trapping of insulin. Higher glucose concentration triggered the faster release of a larger amount of insulin. The microgels were degraded in the presence of dithiothreitol (DTT), which has similar function to glutathione tripeptide (GSH). As a result, a higher amount of insulin was released (90%) from the multiresponsive microgels in the medium (Figure 9b). The degradable multifunctional microgels may provide a new strategy for the design of PBA-functionalized self-regulated drug delivery systems.

**Figure 10:**
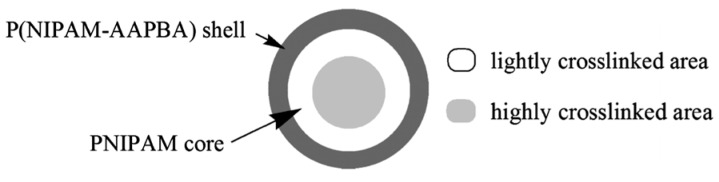
Schematic illustration of the structure of PNIPAM (core)/P(NIPAM-AAPBA) (shell) microgel particles, where the PNIPAM core can be further divided as highly cross-linked “core” with BIS-rich and lightly cross-linked “shell” that is BIS-poor. (Reprinted from ref. [70]).

Additionally, core–shell microgels based on NIPAM and PBA were exploited to investigate their glucose sensitivity. PNIPAM (core)/P(NIPAM-AAPBA) (shell) microgels were prepared by the modification of PNIPAM (core)/P(NIPAM-AA) (shell) microgels with 3-APBA [70]. The core–shell microgels exhibited three structure-related phase transitions when heating (Figure 10). The first phase transition was assigned to the P(NIPAM-AAPBA) shell, while the second and the third phase transitions were related to the PNIPAM core. The structure of the PNIPAM core was heterogeneous. The cross-linking density decreased gradually from the core towards the periphery due to the higher polymerization rate of cross-linking monomer BIS than that of the PNIPAM monomer. As a result, the PNIPAM core had a quasi-core–shell-like structure with a “quasi-core” with a higher cross-linking density and a “shell-like” with a lower cross-linking density. The phase transition temperature of the “shell-like” structure was lower than that of the “quasi-core”, providing the two phase transitions of the PNIPAM core. Besides, the core–shell microgels exhibited glucose-triggered swelling due to the PBA moieties on the P(NIPAM-AAPBA) shell.

The glucose-induced shell permeability of core–shell microgels with the same components of NIPAM and PBA was also demonstrated [71]. Differently, the core of P(NIPAM-AAPBA) microgels was cross-linked by degradable cross-linker *N*,*N′*-(1,2-dihydroxyethylene)bisacrylamide (DHEA), while the shell was cross-linked by BIS. The cross-linked core of the microgels could be degraded by addition of stoichiometric amount of NaIO_4_. The permeability of the P(NIPAM-PBA) shell was controlled by temperature and pH change. More importantly, glucose concentration also could tune the shell permeability. The shell permeability increased with increased glucose concentration, which resulted from the increased borate ester. The fundamental understanding of the glucose-induced permeability control is very important for the design and potential application of PBA-based glucose-sensitive microgels.

PBA-functionalized cross-linking microgels exhibit glucose-induced swelling or shrinking depending on the structure of borate ester between PBA and glucose. At lower glucose concentration, the glucose–bis(boronate) complexes induce the shrinking of microgels with a swollen state. In this condition, there is contraction-type glucose sensitivity. At higher glucose concentration, glucose–mono(boronate) complexes lead to glucose-induced expansion of microgels. Even though the investigations were performed with in vitro studies, it is still worth emphasizing that the reversible glucose-triggered swelling/shrinking changes of PBA-based microgels have potential application in self-regulated drug delivery.

## 4. PBA-Functionalized Nanogels

Even though microgels are most commonly used as the glucose-sensitive drug delivery carrier, the current studies are achieved with the VPTT and in vitro drug delivery. In contrast, nanogels with cross-linking structure are more stable and have received much more attention in drug delivery systems [72]. Nanogels, nanosized particles with cross-linking polymer networks, combine the properties of both hydrogels and nanomaterials [73]. Nanogels have enhanced stability with a long blood circulation time because the particle size and surface properties can be manipulated to avoid rapid clearance by phagocytic cells [74,75]. In addition, a larger surface area of the nanogel is convenient for functionalization of the nanogel [76].

There are several methods for the preparation of glucose-sensitive nanogels based on PBA. Li prepared glucose-sensitive nanogels by one-pot copolymerization of polyethylene glycol methylacrylate (PEGMEM) and AAPBA with BIS as a cross-linking agent [77]. Hollow nanogels composed of PNIPAM and poly(*N*-phenylboronic acid acrylamide) were obtained by two-step colloidal template polymerization [78]. The nanogels had interpenetrating polymer network structure which provided the stability of the nanogels. The morphological structure of inner cavity increased the drug loading content of the nanogels. More importantly, the nanogels were both temperature- and glucose-responsive.

**Figure 11:**
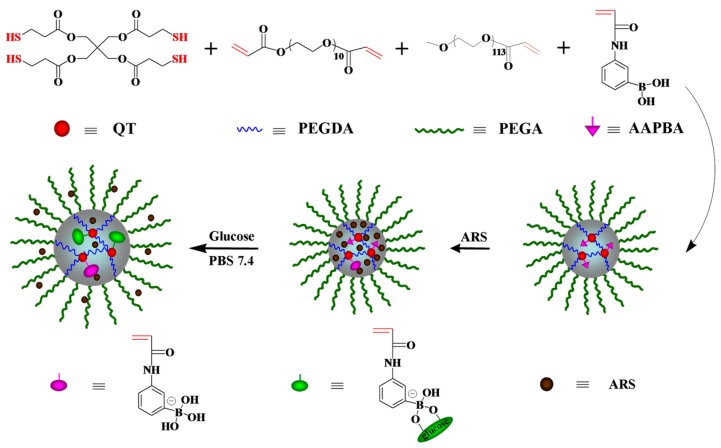
Structure of the nanogel and glucose-sensitive behavior of ARS-loaded nanogel in PBS at pH 7.4. (Reprinted from ref. [79]).

By one-pot copolymerization of pentaerythritoltetra (3-mercaptopropionate) (QT), poly(ethylene glycol) diacrylate (PEGDA), poly(ethylene glycol) acrylate (mPEGA), and AAPBA, a nanogel with a disulfide cross-linked core and PEG shell was obtained [79]. The copolymerization of the monomers was performed via thiol-ene click reaction between thiol and double bond. The obtained nanogels had a core–shell structure. The core was performed by tetrathiol functional QT and bifunctional PEGDA with the free mercapto groups in the nanogel, and the free mercapto groups were terminated by AAPBA and mPEGA (Figure 11). The introduction of PBA group endowed the nanogels with remarkable glucose sensitivity, which was confirmed by fluorescence spectrometry using Alizarin red S (ARS) as a fluorescent probe. Insulin, a model drug, was released from the nanogels with highly glucose concentration dependence. Additionally, methyl thiazolyl tetrazolium, lactate dehydrogenase, and hemolysis assays confirmed the nontoxicity and biocompatibility of the nanogels. The glucose-sensitive nanogel with good biocompatibility has promising potential for application in self-regulated drug delivery.

Nondegradable materials are unfavorable for blood clearance after drug delivery, which limits their clinical application in self-regulated drug delivery. The glucose-sensitive nanogels with improved biocompatibility and degradability have attracted more interest. A novel multifunctional chitosan and PBA-based nanohydrogel with enhanced glucose sensitivity was designed, which was prepared by the modification of chitosan-poly (acrylamide-*co*-methacrylic acid) nanohydrogel with 3-APBA [80]. The glucose-triggered volume phase transition and release profile of the model drug ARS (comparative to insulin as a drug as well as a dye for bioseparation) were studied at various glucose concentrations, pH, and ionic strengths. The nanohydrogel may find applications in bioseparation and glucose-induced drug delivery with enhanced sensitivity toward glucose.

**Figure 12:**
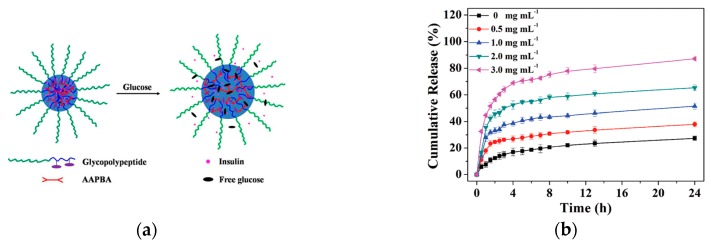
(**a**) Schematic illustration of glucose-triggered insulin release from PBA-functionalized polypeptide nanogel; (**b**) Cumulative insulin release from the insulin-loaded nanogel in PBS with various glucose concentrations at pH 7.4, 37 °C. (Reprinted from ref. [81]).

Besides chitosan, polypeptides are greatly used in drug delivery systems. Chen and coworkers exploited a novel kind of glucose-sensitive polypeptide nanogels via a two-step procedure. The glycopolypeptide, methoxy poly(ethylene glycol)-*block*-poly(γ-benzyl-l-glutamate-*co*-(γ-propargyl-l-glutamate-graft-glucose) (mPEG-*b*-P(BLG-*co*-(PLG-g-Glu))) was fabricated by clicking 2′-azidoethyl-O-*α*-d-glucopyranoside to the PLG unit in mPEG-*b*-P(BLG-*co*-PLG). Then, the mPEG-*b*-P(BLG-*co*-(PLG-*g*-Glu) was cross-linked through boronate esters between the glucose moieties on glycopolypeptide with adipoylamidophenylboronic acid resulting in the glucose-sensitive polypeptide nanogels [81]. Insulin, a model drug, was loaded in the nanogels and the insulin release from the nanogels possessed excellent glucose sensitivity (Figure 12). There was a competitive binding mechanism for glucose-triggered insulin release. When free glucose was added, the complexes between PBA and glucose moieties on glycopolypeptide were destroyed due to the formed complexes between PBA and free glucose. Therefore, more free glucose entered into the nanogel core and the cross-linking density of the nanogels decreased, endowing the nanogels with more hydrophilicity and swelling. As a result, the preloaded insulin was released, induced by glucose, and high glucose concentration triggered more insulin release with a higher release rate. In addition, the polypeptide nanogels exhibited good cytocompatibility and hemocompatibility. The biocompatible nanogels with intelligent glucose-induced insulin release ability may have potential applications in diabetes therapy.

**Figure 13:**
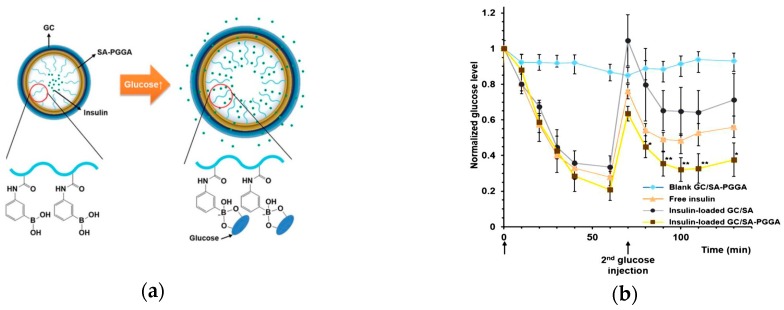
(**a**) Schematic illustration of glucose-sensitive GC/SA-PGGA double-layered nanogel controlled insulin release by complexation between PBA derivatives and glucose; (**b**) Normalized blood glucose levels in mice by retro-orbital administration of blank GC/SA-PGGA nanogels, free insulin (0.5 IU kg^−1^), insulin-loaded GC/SA (0.5 IU kg^−1^), and insulin-loaded GC/SA-PGGA nanogels (0.5 IU kg^−1^). (Reprinted from ref. [82]).

Also using polypeptide, glycol chitosan (GC)/sodium alginate(SA)-poly(l-glutmate-*co*-*N*-3-l-glutamylphenylboronic acid) (PGGA) graft polymer (GC/SA-PGGA) double-layered nanogels were prepared by an isotropic gelation method and electrostatic interactions between GC and SA-PGGA (Figure 13a) [82]. In glucose solution, the binding between glucose molecules and PBA moieties on PGGA converted the PGGA chains to hydrophilic structures. Therefore, the hydrophilicity of nanogels was enhanced, resulting in the swelling of nanogels and consequent insulin release triggered by glucose. Furthermore, a mouse study was conducted to demonstrate the controlled insulin release capability of GC/SA-PGGA double-layered nanogel in vivo. Glucose was administrated to mice by retro-orbital injection to raise the blood glucose levels to diabetic glucose ranges at 0 min. Excluding the blank GC/SA-PGGA group, other groups were administrated glucose again at 70 min (Figure 13b). The blood glucose levels of the groups (excluding blank GC/SA-PGGA group) possessed a similar behavior with progressively lowered glucose levels before the second glucose injection. However, after the second glucose injection, the blood glucose levels of the free insulin and insulin-loaded GC/SA groups no longer had significant decrease. In contrast, the blood glucose level of insulin-loaded GC/SA-PGGA nanogels group was kept relatively low for a longer time, indicating that the nanogels exhibited controlled insulin release with high pharmacological activity to decrease the blood glucose levels. The glucose-triggered insulin release makes the GC/SA-PGGA double-layered nanogels a promising approach for diabetes treatment.

**Figure 14:**
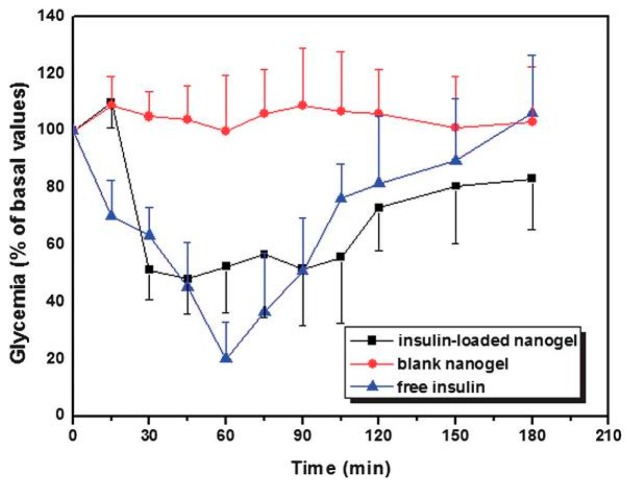
Profiles of glycemia after a subcutaneous administration of free insulin (2.0 IU kg^–1^), insulin-loaded nanogels (4 IU kg^–1^), and blank nanogels in fed diabetic rats. Before the injections, glycemia was 426 ± 13 mg mL^–1^. Results are expressed as mean ± SD (*n* = 8). (Reprinted from ref. [83]).

An injectable nanogel with interpenetrating polymer networks of PNIPAM, dextran, and poly(3-acrylamidophenylboronic acid) (P(NIPAM-Dex-PBA)) was exploited using maleic acid-dextran as a cross-linker [83]. The nanogels presented reversible glucose sensitivity under physiological conditions, which was related to dextran content. The preloaded insulin was released from the nanogels with high dextran content triggered by glucose. More importantly, the considerable hypoglycemic effect of the insulin-loaded nanogels was also confirmed. As shown in Figure 14, in vivo experiments demonstrated that the blood glucose level of diabetic rats treated with insulin-loaded nanogels was maintained in a low state for almost two hours. The reduction of blood glucose level for insulin-loaded nanogels treated diabetic rats was 51% of the baseline level, which was associated with long-term controlled insulin release triggered by glucose. In addition, insulin-loaded nanogels maintained stable blood glucose levels without remarkable fluctuations of blood sugar. The insulin-loaded nanogels with prolonged and stable blood glucose reduction effect may have potential applications for diabetes treatment.

**Figure 15:**
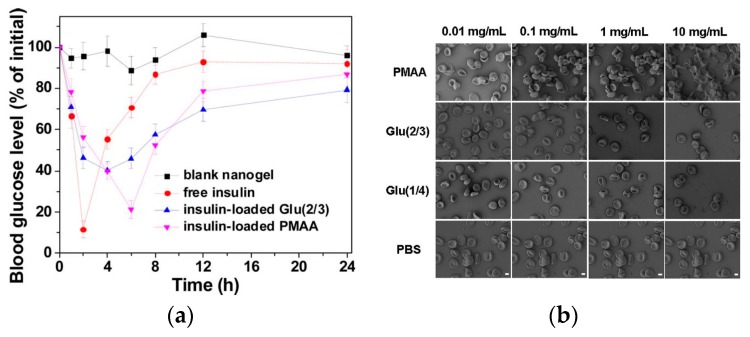
(**a**) The profiles of rats’ blood glucose concentrations after injection with different samples; (**b**) Effect of the different nanogels on the aggregation and morphology of red blood cells. (Reprinted from ref. [84]).

Even though most PBA-functionalized nanogels are exploited to achieve the glucose sensitivity and glucose-triggered hypoglycemic effect, the particular and comprehensive studies on the blood compatibility of the nanogels have been reported rarely. Zhou and coworkers explored the glucose-sensitive insulin controlled release and blood compatibility of PBA-based nanogels [84]. The nanogels were prepared through a thermally initiated precipitation copolymerization of methacrylic acid (MAA) and AAPBA monomers using ethylene glycol dimethacrylate (EGDMA) as a cross-linker. By changing the molar ratio of MAA/AAPBA, nanogels with different PBA contents were obtained and noted as Glu(a/b), in which a/b represent the molar ratio of MAA/AAPBA. The release of FITC-insulin from Glu(2/3) was dependent on the glucose concentrations without initial burst release. In particular, the blood glucose levels of diabetic rats treated with insulin-loaded Glu(2/3) nanogels were maintained at a low concentration (below the 70% of initial blood glucose level) with a long-term stable hypoglycemic effect compared with the free insulin group (Figure 15a). Moreover, a series of blood assays and in vitro/vivo assays confirmed the good blood compatibility of PBA-based nanogels. The nanogels did not cause aggregation and morphological change of the red blood cells (Figure 15b). The activated partial thromboplastin time (APPT) is used to evaluate the intrinsic and common coagulation pathways. The glucose-sensitive nanogels had no interactions with the coagulation factors and/or partial thromboplastin reagent which was confirmed by APTT. In addition, the nanogels had little effect on the extrinsic pathway of blood coagulation examined by prothrombin time (PT). Thromboelastography (TEG) assay also indicated the anticoagulant effects which were attributed to the removal of thrombin from the blood by the nanogels via electrostatic interactions. Toxicity studies further confirmed the blood compatibility of the nanogels. This work provides a strategy for the study of biocompatibility of glucose-sensitive drug carriers. Thus, compatible nanogels with good hypoglycemic effect have potential application in diabetes therapy.

## 5. Conclusions

PBA-functionalized hydrogels, microgels, and nanogels provide glucose-triggered drug release due to reversible swelling and shrinking induced by glucose. PBA-based glucose-sensitive hydrogels can be prepared by physical or chemical cross-linking, such as the self-aggregation of gelators, cross-linkage by cross-linkers, or reversible covalent complexation of PBA with *cis*-1,2 or *cis*-1,3-diol compounds. Even though the PBA-mediated hydrogels possess great glucose sensitivity, the poor ease of administration, i.e., they cannot be administrated by injection but probably as an implant, limit their application in diabetes therapy. In contrast, PBA-based microgels and nanogels with micro- or nano-scale dimensions exhibit excellent biocompatibility, tunable sizes, large bioconjugated surfaces, as well as convenient injection administration. For PBA-based microgels, studies are focused on the VPTT and the mechanism of glucose sensitivity. The development of PBA-based nanogels has seen significant progress in terms of glucose sensitivity, biocompatibility, and pharmacokinetic and hypoglycemic effect. PBA-functionalized nanogels with convenient administration via injection have practical application for diabetes therapy.

Even though there is considerable progress of PBA-based hydrogels, microgels, and nanogels for glucose-sensitive drug delivery, some challenges still have to be overcome to promote the clinical application of PBA-mediated platforms in diabetes therapy.

Firstly, the specificity and selectivity of glucose sensitivity are important for the design and application of glucose-sensitive platforms. Besides glucose, there are physiologically relevant saccharides, such as fructose, mannose, and galactose, which can bind to PBA and form mono(boronate) complexes. PBA and derivatives are able to form mono(boronate) complexes as well as bis(boronate) complexes with glucose. The influence of sugars on glucose sensitivity of PBA-based platforms must be considered. Additionally, the glucose sensitivity of drug carriers must be selective, that is, the platforms must timely and rapidly adjust the insulin release on-demand. The ideal drug carrier should release insulin quickly with precise dosage in response to hyperglycemic state, while not releasing glucose during normoglycemic states to keep the blood glucose levels in the normal range.

Secondly, adopting simplified preparation of PBA platforms is conducive to maintain the repeatability and controllability of the structure and property of glucose-sensitive carriers for different preparations. In addition, the glucose-sensitive platforms with high bioactivity of preloaded insulin should be administrated easily. Injection is an ideal approach because it can reach blood circulation with enhanced glucose sensitivity. The drug delivery system must be designed ingeniously with easy and repeatable preparation and convenient administration to promote its clinical application.

Thirdly, biocompatibility without long-term side effects of the platforms is a challenge for the design of PBA-based glucose-sensitive drug delivery systems. Diabetes is a lifelong chronic disease and the treatment of diabetes is a long-term process. Therefore, glucose-sensitive platforms must be nontoxic and friendly to the body without inducing inflammation. By adopting biodegradable and biocompatible materials, such as poly(acrylic acids) and polypeptides, the biocompatibility of PBA-based matrices can be enhanced.

Although some issues still need to be overcome, self-regulated drug delivery systems have promising application in diabetes treatment.
